# Understanding the predictive accuracy of the InsuTAG index over other surrogate indices in normoglycaemic, non-obese males from Southern India

**DOI:** 10.1038/s41598-023-45880-z

**Published:** 2023-11-09

**Authors:** Felix K. Jebasingh, Shajith Anoop, Riddhi Dasgupta, Mathews Edatharayil Kurian, Aneez Joseph, Grace Rebekah, Venkataraghava Mohan, Nihal Thomas

**Affiliations:** 1 Department of Endocrinology, Diabetes and Metabolism, Christian Medical College Vellore, Ida Scudder Road, Vellore, Tamilnadu 632004 India; 2 Department of Biostatistics, Christian Medical College, Vellore, India; 3 Department of Community Health and Development, Christian Medical College, Vellore, India

**Keywords:** Physiology, Endocrinology, Health care, Medical research

## Abstract

We aimed to evaluate the predictive accuracy of InsuTAG index against *M* value of the hyperinsulinaemic-Euglycaemic clamp (HEC) procedure and fasting surrogate indices of insulin sensitivity/resistance in young, normoglycaemic, Asian Indian males. HEC studies were done in young (mean age 19.7 ± 1 years), non-obese (mean BMI 19.2 ± 2.6 kg/m^2^), normoglycemic Asian Indian males (n = 110) and the *M* value was calculated. Surrogate indices namely InsuTAG index, HOMA-IR, FG-IR, QUICKI and McAuley index were calculated. Pearson’s correlation and ROC-AUC at 95% CI were applied. Significant negative correlation was observed for InsuTAG index with the *M* value (*r* − 0.23, *p* = 0.01), McAuley index (*r* − 0.65, *p* < 0.01), QUICKI (*r* − 0.34, *p* < 0.01) and FGIR (*r* − 0.35, *p* < 0.01). Significant positive correlations of InsuTAG index were observed for BMI and waist circumference. The ROC-AUC was higher for InsuTAG index (0.75) than FGIR (0.30), QUICKI (0.31), and McAuley index (0.20). The InsuTAG cut-off value ≥ 19.13 showed 66.7% sensitivity and 69.2% specificity in this study group.

## Introduction

The prevalence of metabolic syndrome is high in South Asians due to genetic and lifestyle factors^1^. Data from the International diabetes federation (IDF) estimate that 3.5% of Asian Indians aged between 20 and 79 years would develop impaired glucose tolerance by the year 2030^2^. The incidence of diabetes amongst metabolically-obese, non-obese Asian Indians is 41.3% in and 15.9% in metabolically-healthy and non-obese individuals (mean BMI: 20.6 kg/m^2^) aged 37 years and above^3^. Classical surrogate indices have limited predictive accuracy in Asian Indians^4^, thereby implying the need to develop novel indices with better predictive accuracy especially in young populations.

Any surrogate index of insulin resistance or sensitivity needs to be validated against the gold standard prior to its application in a specific population. The hyperinsulinaemic-euglycaemic pancreatic clamp (HEC) procedure is the gold standard used for measuring whole body insulin resistance and to validate a surrogate index^5^. However, it is not feasible to apply the HEC procedure in clinical settings and epidemiological surveys as it is technically cumbersome, requires trained manpower and state-of-art laboratory facilities. In such a scenario, classical surrogate indices of insulin resistance namely the Homeostasis model of assessment for insulin resistance (HOMA-IR), the Quantitative insulin sensitivity check index (QUICKI), the Fasting glucose to insulin ratio (FG-IR) are used routinely in epidemiological studies and clinical settings across various populations^6^. Certain studies have applied surrogate indices for insulin resistance based on fasting plasma glucose and insulin^6,7^ and reported differential results in terms of sensitivity and specificity in Asian Indians^4^.

The triglyceride-based surrogate indices have been reported to have higher predictive accuracy for hepatic and peripheral resistance in certain ethnic groups^8–10^. The InsuTAG index has been reported to have higher predictive accuracy for insulin resistance and metabolic syndrome in obese individuals^11^. However, this index has not been researched in normoglycaemic and non-obese individuals. In this study, we aimed to compare the predictive accuracy of the InsuTAG index against the *M* value of the HEC procedure and with fasting surrogate indices viz HOMA-IR, FG-IR, QUICKI, and the McAuley index. Secondly, we aimed to derive a cut-off value for the InsuTAG index which may be used to predict the risk of insulin resistance in a homogenous group non-obese, normoglycaemic Asian Indian males.

### Research design and methods

This is a cross sectional study based on a homogenous group of non-obese, normoglycemic males. It was approved by the institutional review board (IRB Number: 13348/RETRO/28/08/2020) of Christian Medical College Vellore, India and was conducted in accordance to the ethical principles for medical research stipulated by the declaration of Helsinki, 2013. This study is exclusively based on healthy male individuals who were recruited from the birth registry at the Community Health and Development (CHAD) centre at CMC Vellore. The detailed methodology of this study design has been published earlier^12^. Briefly, the participants of the study were identified from 23 randomly selected villages from the North Arcot District of Vellore, Tamilnadu, Southern India. The contact details of subjects were obtained from the birth registry of the CHAD centre of the institution. The families of 265 individuals from the community were contacted in person and interviewed. Male individuals aged between 18 and 22 years from the families were shortlisted and invited. The objectives of the study were explained to the participants and informed, written consent was obtained from them. Out of 265 individuals, 117 individuals participated in the initial study. Furthermore, individuals with pre-diabetes, impaired fasting glucose and dyslipidemia, any form of infectious disease, chronic alcohol dependence and substance abuse were excluded from participation. In accordance to the objectives of the current study, the sample size was calculated as 113 subjects with absolute precision of 90% with an expected proportion of 0.75% at 95% confidence interval. A total of 110 individuals were recruited. All participants underwent baseline biochemical assessment and a standard oral glucose tolerance test (OGTT) with 75 g dextrose. Normoglycaemic individuals as determined on the OGTT were recruited for HEC studies. The detailed methodology of the OGTT is published in a previous study from the same group^12^. To account for the effect of gender, this study was designed to study insulin sensitivity in non-obese, young males exclusively. Therefore, females were not recruited in the primary study as per protocol.

### Anthropometry and DEXA imaging

All the participants were monitored by the physician throughout the study period. The participants underwent a clinical examination. On the first day, the participants underwent a physical examination for height, weight, blood pressure and skinfold thickness measurements of biceps and triceps. Whole body composition was analysed by non-invasive, non-hazardous, Dual energy X-ray absorptiometry (DXA ; QDR 4500, Hologic, Inc, Waltham, USA). The total lean mass, fat-free mass, fat mass and body mineral density were estimated. Bilateral sections and whole-body composition data were obtained by analysis of the regions of interest (ROI) using APEX software (Version 4.0.2).

### Hyperinsulinaemic-Euglycaemic pancreatic clamp (HEC) procedure

The participants were explained about the HEC procedure and informed consent was obtained. On the day of HEC procedure, the participants reported to the laboratory in fasting state and the vital parameters were assessed. An indwelling catheter was inserted contra-laterally in the veins of the antecubital fossa of the left arm and a continuous insulin infusion was initiated by using an infusion pump. The insulin flow rate was maintained at 40 mU m^−2^ per minute during the entire duration of the 2 hour HEC procedure. The second catheter was inserted on the veins of the antecubital fossa of the right arm and used for blood draw. The blood draw lines were kept patent by maintaining them at 65 degrees centigrade by placing the arm over prewarmed, sterile saline bags wrapped in a cotton case. To maintain euglycaemia [90 mg/dl i.e. 5 mmol/l] throughout the HEC procedure, a 25% intravenous dextrose solution was infused with meticulously adjusted flow rates and plasma glucose levels were measured every 5 min using a bedside glucose analyser (Analox GM-9D). Blood samples were drawn for the estimation of insulin, and C-peptide levels at baseline and at the end of the steady state (i.e. last 30 min of the basal phase and the last 30 min of the clamp period). The Serum insulin and C-peptide levels were measured by the Chemiluminescence method on the Immulite 2000 system by using commercial kits (Siemens healthcare Diagnostic products Ltd, Llanberis, Gwynedd, UK). Immunoassay controls supplied by Bio-Rad were used as internal precision controls. The coefficient of variation (CV) was 10.2% for insulin and 3.7% for C-peptide. Plasma glucose levels were measured by the glucose-oxidase method. The serum lipid profile was measured using an enzyme based colorimetric method in an automated analyzer (COBAS-B, 101 system, Roche Diagnostics Ltd).

### *M* value

The *M* value is a measure of whole body insulin sensitvity. In other words, the *M* value denotes the rate of whole body glucose metabolism at a single level of hyperinsulinemia during a pancreatic clamp procedure. It is ideal to calculate the *M* value from the dextrose infusion rate maintained between 60 and 120 min after the start of the insulin infusion in a HEC procedure. At this time interval the coefficient of variation for plasma glucose, insulin and glucose levels is the lowest^13^. To achieve a “steady state” of hyperinsulinemia, a continuous infusion of exogenous insulin was maintained at a constant rate of 40 mU m^−2^ per minute for 120 min. The steady state was calculated between 60 and 120 min after the start of the insulin infusion, based on the formula of Defronzo *et al.*^5^. The *M* value is calculated by the formula.

*M* = *GIR-SC-UC* wherein GIR is the rate of glucose infused exogenously, SC is the space correction (space correction indicates the changes in glucose infusion rate adjusted according to changes in plasma glucose levels during the HEC procedure) and UC is the correction done for loss of glucose through urine. The value of *SC* is calculated for every 20 min during the HEC procedure using the formula;

$$SC\left( {{\text{mg}}\, {\text{kg}} \,{\text{min}}^{{ - {1}}} } \right)\, = \,\left( {G2\, - \,G1} \right)\, \times \,{1}0\, \times \,(0.{19}\, \times \,{\text{body}}\,{\text{ weight }}\left( {{\text{kgs}}} \right)/{2}0\, \times \,{\text{body }}\,{\text{weight}}\, \, ({\text{kgs}}).$$

*SC* (mg kg min^−1^) = (*G2* − *G1*) × 0.095 wherein G1 and *G2* are the initial and final glucose levels respectively during a HEC procedure. The multiplication of (*G2 − G1*) with the factor 10 in the numerator converts the units of plasma glucose levels from mg/dl to mg/litre. Furthermore, the difference in plasma glucose levels (in mg/litre) is multiplied with the whole-body distribution volume of glucose expressed in litres [0.19 (litre/kg body-weight) and further multiplied with body weight (kgs). By cancelling the litres in the numerator, the difference in the whole-body glucose metabolism between the beginning and the end of the 20 min time period is derived. The division of this difference in glucose level (mg) by the denominator term (20 min x body weight) accounts for time (i.e. 20 min) and body weight (kg) and finally converts the dimension to mg^.^kg^-1.^min^-1.^ The *M* value is normalised for fat free mass of an individual and therefore expressed as mg/kg^−1 ^min^−1^^14^.

As there are no cut-off values to define insulin resistance using HEC procedures in Asian Indians, we referred to the value < 4.7 mU m^−2^ per minute on the HEC procedure determined previously in 18 HEC studies in different ethnic groups by Bergman et al. All the HEC procedures used a constant insulin infusion rate of 40 mU m^2^ per minute^15^ which is identical to the current study. In addition, the following surrogate indices of insulin resistance/sensitivity based on fasting insulin, glucose and triglycerides were calculated by using specific formulae;

- InsuTAG index: Fasting serum insulin (uU/ml) × Fasting Triglyceride levels (mmol/L)^11^
- HOMA-IR: Fasting glucose (mmol/L) × fasting insulin (mU/L)/22.5^7^.
- QUICKI: 1/[log fasting insulin (mU/L) + log fasting glucose (mg/(mg/dL)]^16^.
- FG-IR: Fasting glucose (mg/dL)/fasting insulin (mU/L)^17^
- McAuley index : exponential of the product of {2.63–0.28 × [fasting serum insulin (IU/mL)]-0.31 × [serum TG (mmol/L)^18^.
- Triglyceride-HDL-C ratio: Triglycerides (mg/dl)/HDL-C (mg/dl)^19^

### Statistical analysis

Data was checked for Normality by applying the Shapiro–Wilk test. Continuous variables were summarized as Mean ± SD/median values as appropriate. Pearson’s partial correlation was applied to test the significance in correlation between the surrogate indices of insulin resistance and the *M* value. Stepwise multiple linear regression analysis was applied to derive the significant determinants of the InsuTAG index. Receiver Operator characteristics (ROC) analysis with Area Under the Curve (AUC) was applied to determine the sensitivity and specificity of the surrogate indices at 95% confidence interval. The *p* value < 0.05 was considered statistically significant. SPSS Version 21.0 was used for data analysis. All authors complied with the ARRIVE guidelines.

## Results

The participants belonged to a rural population of South India. Of the 110 participants, 54. 5% (n = 60) and 45.4% of them (n = 50) were born with normal birth weight and low birth weight respectively. Participants with low birth weight were shorter (167.3 ± 6.8 cm) and of less total body weight (52.9 ± 8.6 kgs) when compared to those born with normal birth weight (171.6 ± 6.1 cms, total body weight: 57.8 ± 8.2 kgs). The mean *M* value in this study group (10.5 ± 3.8 mg/kg^−1 ^min^−1^) showed higher degree of insulin sensitivity in the study group (Table 1). With reference to *M* value cut-off value determined from HEC studies by Bergman et al.^15^, the proportion of subjects with insulin resistance (*M* value < 4.7 mg/kg/min) was significantly lower (*n* = 3; 2.7%) when compared to those who were insulin sensitive (*M* value ≥ 4.7 mg/kg/min) (*n* = 107; 97.3%). The participants were non-obese with normal waist circumference. Amongst biochemical variables, the mean values of total cholesterol, LDL-C and HDL-C were in normal range. However, the standard deviation for serum triglycerides and LDL-C were higher. Amongst triglyceride-based surrogate indices, the mean and median value of the InsuTAG index was higher than the triglyceride/ HDL ratio as shown in Table 1.

**Table 1:** Anthropometry and metabolic profile of the study group.

Variables (n = 110)	Mean ± SD/Median
Age (years)	19.7 ± 1.0
BMI (kg/m^2^)	19.2 ± 2.6
Waist circumference (cm)	70.1 ± 7.0
Waist-to-hip ratio	0.83 ± 0.04
Fasting glucose (mg/dL)	88.2 ± 5.4
Fasting Insulin (pmol/L)	4.3 ± 3.5* (2.1, 5.4)
Fasting C-peptide (ng/ml)	1.7 ± 1.1*(0.7, 1.9)
Post prandial blood glucose (mg/dL)	100.8 ± 21.6
Post prandial Insulin (pmol/L)	36.7 ± 29* (15.1, 47.3)
Post prandial C-peptide (ng/ml)	5.1 ± 2.9* (2.6, 7)
Total cholesterol (mg/dL)	127.4 ± 27
Low density lipoprotein cholesterol (mg/dL)	77.2 ± 19.3
High density lipoprotein cholesterol (mg/dL)	30.8 ± 3.8
Triglycerides (mg/dL)	78 ± 26.5
Indices of insulin sensitivity/ resistance	
*M* value (on HEC procedure) (mg/kg FFM per min)	10.5 ± 3.8
InsuTAG index	18.4 ± 13.6 *(7.3, 25.5)
HOMA-IR	0.94 ± 0.8* (0.4, 1.2)
QUICKI	0.4 ± 0.06
Fasting glucose-insulin ratio	36.7 ± 24* (15.7, 41.7)
McAuley index	6.7 ± 1.8
Triglycerides /HDL ratio	2.5 ± 2.3* (1.7, 3.3)

Median values are indicated by * with 25th and 75th Quartile shown in parentheses.

*FFM* fat free mass.

Pearson correlation demonstrated significant but a lower degree of negative correlation of the InsuTAG index with the *M* value (*r* − 0.23, *p* = 0.01). Amongst insulin based surrogate indices of insulin resistance, significant negative correlation was observed for the InsuTAG index with the McAuley index (*r* − 0.65, *p* < 0.01,) the QUICKI (*r* − 0.34, *p* < 0.01) and the FGIR (*r* − 0.35, *p* < 0.01). No significant correlation was observed for the InsuTAG index with the HOMA-IR. As for anthropometric measures, a significant positive correlation was observed for the InsuTAG index with BMI and WC as shown in Table 2.

**Table 2:** Correlation of the InsuTAG index with measures of insulin sensitivity/ resistance and anthropometric indices.

Measures of insulin sensitivity/ resistance	*r* value	*P* value
*M* value (measure of whole-body insulin sensitivity determined in a HEC procedure)	− 0.23	**< 0.05**
McAuley Index	− 0.65	< 0.01
Fasting glucose-insulin ratio	− 0.35	< 0.01
QUICKI	− 0.34	< 0.01
HOMA-IR	0.77	0.44
Anthropometric indices		
Body mass Index (Kg/m^2^)	0.29	< 0.01
Waist circumference (cm)	0.38	< 0.001
Waist-to-hip ratio (WHR)	0.18	0.05

*P* value < 0.05: Statistically Significant.

**Figure 1:**
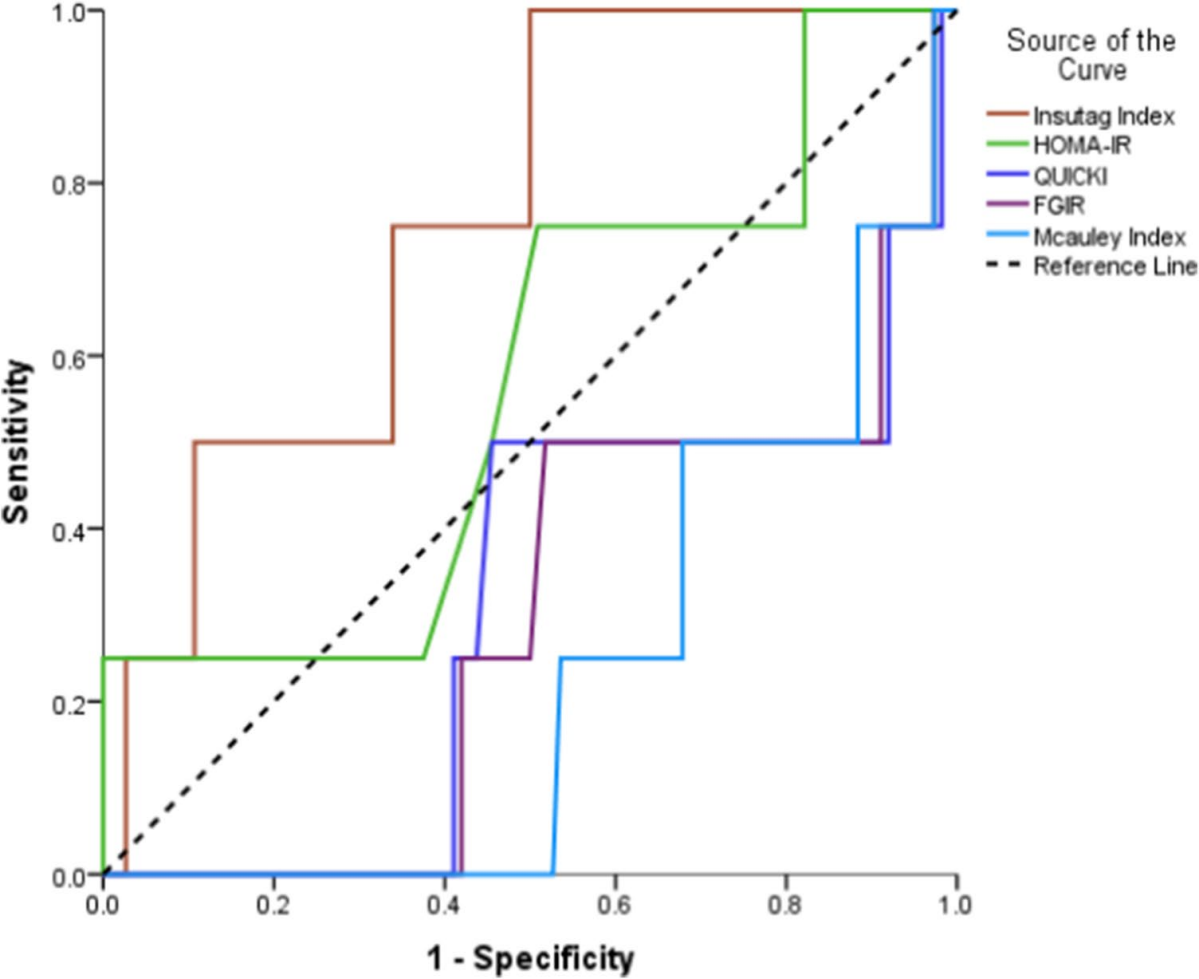
ROC Area under curve (AUC) for InsuTAG and other surrogate indices.

The ROC-AUC was higher for the InsuTAG index when compared to the FGIR and the QUICKI but not statistically significant as shown in Table 3**.** The ROC-AUC for all indices is shown as Fig. 1.

**Table 3:** ROC analysis with area under curve (AUC) for different surrogate indices.

Surrogate Indices	Area under curve	95% CI	SE	*P* value
InsuTAG index	0.75	0.56, 0.95	0.10	0.08
HOMA-IR	0.57	0.28, 0.86	0.15	0.63
QUICKI	0.31	0.04, 0.57	0.13	0.20
FG-IR	0.30	0.05, 0.54	0.13	0.17
McAuley index	0.23	0.05, 0.41	0.09	0.07

*P* value < 0.05: Statistically significant: *CI* confidence interval, *SE* standard error.

In this study, the cut-off value ≥ 19.13 for the InsuTAG index achieved 66.7% sensitivity and 69.2% specificity at an AUC of 0.75. [Positive Predictive Value (PPV): 5.7%, Negative Predictive Value (NPV): 98.7%, Youden’s index: 0.46 and Positive likelihood ratio of 2.1. This implies that a young, non-obese, normoglycemic Asian Indian male who scores ≥ 19.13 on the InsuTAG index has 5.7% probability of developing insulin resistance in the later years of his life. The sensitivity was 66.7% for other indices namely the FG-IR, the HOMA-IR, the QUICKI and the McCauley index with differential specificity for each index. It was highest for the InsuTAG index followed by other indices as shown in Table 4.

**Table 4:** Sensitivity, specificity and predictive values of the surrogate indices.

Surrogate Indices	Cut-off value	Sensitivity (%)	Specificity (%)	PPV (%)	NPV(%)	LR+	LR–
InsuTAG index	≥ 19.13	66.7	69.2	5.7	98.7	2.16	0.48
HOMA-IR	≥ 0.75	66.7	52.3	3.8	98.2	1.4	0.63
FG-IR	≥ 23.4	66.7	45.8	3.3	98.0	1.2	0.73
QUICKI	≥ 0.40	66.7	54	3.4	98.2	1.1	0.58
McAuley Index	≥ 5.71	66.7	29.0	2.6	97	0.94	1.15

*PPV* positive predictive value, *NPV* negative predictive value, *LR*+ positive likelihood ratio, *LR*− negative likelihood ratio.

On regression analysis, fasting insulin, HDL and the TG/HDL ratio were derived as significant determinants of the InsuTAG index (regression coefficient (*r*^2^): 0.65 as shown in Table 5.

**Table 5:** Stepwise multiple linear regression analysis for the InsuTAG Index.

Determinants of the InsuTAG index	β coefficient	95% CI	SE	*P* value
Fasting insulin (pmol/l)	3.2	(3.4, 4.1)	0.17	< 0.01
Triglycerides/HDL ratio	7.4	(6.3, 8.4)	0.53	< 0.01
HDL (mg/dl)	0.35	(0.17, 0.53)	0.09	<0.01

*P* < 0.05: Statistically significant, *CI* confidence interval, *SE* standard error.

## Discussion

With the increasing prevalence of insulin resistance, earlier onset of diabetes mellitus and cardiovascular diseases even in non-obese individuals, it is important to develop simple and accurate indices that may be used in clinical settings and epidemiological studies^6^ to screen individuals for the risk of insulin resistance. In the recent years, the InsuTAG index has garnered much interest as it combines insulin and triglycerides as biochemical parameters to evaluate for risk of insulin resistance in an individual. Previous studies have reported that surrogate indices based on fasting triglycerides levels have higher sensitivity and specificity for predicting the risk of insulin resistance in comparison to other surrogate indices (11)^20^. Notably, there are no studies which have validated the InsuTAG index against the HEC procedure and other surrogate indices in a homogenous cohort of normoglycemic individuals from a single ethnic group. This is the first single centre study from India wherein the predictive accuracy of the InsuTAG index has been evaluated against the *M* value derived from HEC procedures (the gold standard measure of Insulin sensitivity) and other glucose and insulin based surrogate indices of insulin resistance in a homogenous group of young, normo-glycaemic males from a rural population of Southern India. As this study is exclusively on a single group of non-obese, normoglycemic individuals, individuals with impaired glucose tolerance or diabetes were not included. Our previous studies in the same group have consistently shown higher sensitivity and specificity of triglyceride-based indices. In an earlier study, we reported higher predictive accuracy for the triglyceride/glucose ratio over HOMA-IR, FG-IR, QUICKI, and the McAuley index based on observations from HEC studies in normoglycemic males^21^. Subsequently, in another study we also reported higher predictive accuracy of the Lipid accumulation product index (a triglyceride based index) over HOMA-IR, QUICKI and FG-IR in non-obese, normoglycemic males^22^.

The common method of evaluating the predictive accuracy of surrogate indices is by using correlation coefficients against a gold standard^23^ and applying the Receiver Operator characteristics (ROC) analysis with Area Under Curve (AUC)^24^. The terms sensitivity and specificity are used commonly to assess the performance of a diagnostic index against a gold standard^25^. Sensitivity refers to the proportion of subjects who are tested positive using the diagnostic index. Specificity is the accuracy with which the diagnostic index differentiates individuals with true disease from individuals without the disease status. Sensitivity is inversely related to specificity. The plot of sensitivity versus 1-specificity is statistically calculated as Receiver Operator Characteristics (ROC) with Area under the Curve (AUC) for statistical interpretations about the accuracy of index or a marker^26^. The positive predicted values (PPV) and the negative predicted values (NPV) are two other parameters that are used in a diagnostic index. The PPV is defined as the probability of an individual actually having the disease or disorder and the NPV is defined the probability of an individual being truly healthy for negative test results on the diagnostic index^25^.

We applied the ROC-AUC analysis and noted that the predictive accuracy of the InsuTAG index did not differ significantly when compared to the HOMA-IR, the FG-IR, the QUICKI and the McAuley index. We correlated the InsuTAG index with the *M* value and observed an inverse correlation. This can be attributed to higher insulin sensitivity in the study subjects of this group (as indicated by higher mean *M* value). Of note, the study participants in this study were young, normoglycemic individuals with ethnic and phenotypic homogeneity and higher insulin sensitivity in contrast to the former study^11^ which included elderly and obese insulin resistant individuals of three different ethnic groups.

In this study, the cut-off value ≥ 19.13 for the InsuTAG index attained 66.7% sensitivity and 69.2% specificity which are comparatively lower that the sensitivity and specificity values determined for InsuTAG index in an earlier study in elderly Caucasian subjects^11^. This is evident as study subjects in the former study^11^ were elderly and obese with metabolic syndrome and insulin resistance in contrast to the current study in young, non-obese, normoglycemic males without metabolic syndrome. With reference to the PPV (5.7%) and NPV (98.7%) of the InsuTAG index in the current study, it can be stated that 98.7% of those individuals who score < 19.13 on the InsuTAG index do not have insulin resistance. In other words, it can be estimated that only 5.7% individuals of this group who score ≥ 19.13 on the InsuTAG index may develop Insulin resistance in the later stages of their life. These observations are in contrast to the study on InsuTAG Index by Thota et al.^11^, wherein the specificity (84.1%) and sensitivity (86.8%) were significantly higher for the InsuTAG index cut-off value ≥ 11.2 in a group of obese and elderly individuals with metabolic syndrome and different BMI values. In contrast to the former, the current study on InsuTAG index was done on a group of young, non-obese and normoglycemic individuals with a mean triglyceride level that was comparatively lower than the former^11^. Therefore, the difference in cut-off values for the InsuTAG index is evident. In the former study^11^, binary logistic regression analysis was applied in a heterogenous group and variables such as age, gender, waist circumference and C-reactive protein were the significant determinants of the InsuTAG index. However, in the current study, stepwise multiple linear regression analysis was applied as the participants were normoglycemic and phenotypically homogenous thereby deferring the need to classify them into different study groups.

The significant determinants of the InsuTAG index in the current study were insulin, high density lipoprotein cholesterol (HDL-C) and the triglyceride/ HDL-C ratio. The cut-off value for InsuTAG index (≥ 19.13) in the current study was comparatively higher than the cut-off value ≥ 11. 2 determined in the former^11^. The differences in the cut-off values for the InsuTAG index are plausible due to variations in age, BMI, metabolic status, and ethnicity between the two studies. In the previous study in a group of obese and elderly individuals with metabolic syndrome and different BMI values, the cut-off value for the InsuTAG index was derived with reference to HOMA-IR^11^ whereas in the current study, the cut-off value was derived with reference to *M* value obtained from HEC studies, which is the gold standard measure of whole body insulin sensitivity.

In summary, this is the first study from India to validate the InsuTAG index against the HEC studies in normoglycemic individuals. The results of the current study based on HEC procedures in 110 individuals suggest that the InsuTAG index can be used as a better surrogate index to screen for risk of insulin resistance in normoglycemic individuals with low BMI, but not as an index for clinical diagnosis. The cut-off value for the InsuTAG index obtained in the current study can be used as normative reference value for non-obese, normoglycemic Asian Indian males. Furthermore, this is the second study on InsuTAG index after the primary study by Thota et al.

The limitations of the study are acknowledged. This includes the cross-sectional  study design in a group of young, non-obese male individuals. The cut-off value for the InsuTAG index obtained in the current study is applicable only to non-obese, normoglycaemic males in the age group of 18 to 22 years. Therefore, testing the InsuTAG index in prospective studies of representative samples of normoglycaemic males without metabolic syndrome and across BMI ranges is essential to determine age and BMI specific cut-off values. Secondly, the InsuTAG index can be used only in resource clinical settings that have facilities for insulin assays.

## Data Availability

The data used in the study can be made available on requests addressed to the corresponding author.

## References

[1] Jayawardena R, Sooriyaarachchi P, Misra A (2021). Abdominal obesity and metabolic syndrome in South Asians: Prevention and management. Expert Rev. Endocrinol. Metab..

[2] IDF Atlas 9th edition and other resources. https://www.diabetesatlas.org/en/resources/. Accessed 27 Oct 2021.

[3] Natarajan H, Shanthi Rani CS, Krishna Kumar D (2021). Future risk of diabetes among Indians with metabolic and phenotypic obesity: Results from the 10-year follow-up of the Chennai urban rural epidemiology study (CURES-158). Acta Diabetol..

[4] Muniyappa R, Irving BA, Unni US (2010). Limited predictive ability of surrogate indices of insulin sensitivity/resistance in Asian-Indian men. Am. J. Physiol. Endocrinol. Metab..

[5] DeFronzo RA, Tobin JD, Andres R (1979). Glucose clamp technique: A method for quantifying insulin secretion and resistance. Am. J. Physiol..

[6] Muniyappa R, Lee S, Chen H, Quon MJ (2008). Current approaches for assessing insulin sensitivity and resistance in vivo: Advantages, limitations, and appropriate usage. Am. J. Physiol. Endocrinol. Metab..

[7] Katsuki A, Sumida Y, Gabazza EC (2001). Homeostasis model assessment is a reliable indicator of insulin resistance during follow-up of patients with type 2 diabetes. Diabetes Care.

[8] Unger G, Benozzi SF, Perruzza F, Pennacchiotti GL (2014). Triglycerides and glucose index: A useful indicator of insulin resistance. Endocrinol. Nutr. Organo Soc. Espanola Endocrinol. Nutr..

[9] Lucatello F, Vigna L, Carugno M, Tirelli AS, Bertazzi PA, Riboldi L (2012). Comparison of indexes for assessing insulin resistance for the health surveillance among workers. G. Ital. Med. Lav. Ergon..

[10] Vasques ACJ, Novaes FS, de Oliveira M, da S, (2011). TyG index performs better than HOMA in a Brazilian population: A hyperglycemic clamp validated study. Diabetes Res. Clin. Pract..

[11] Thota RN, Abbott KA, Ferguson JJA (2017). InsuTAG: A novel physiologically relevant predictor for insulin resistance and metabolic syndrome. Sci. Rep..

[12] Thomas N, Grunnet LG, Poulsen P (2012). Born with low birth weight in rural Southern India: What are the metabolic consequences 20 years later?. Eur. J. Endocrinol..

[13] Bokemark L, Frödén A, Attvall S, Wikstrand J, Fagerberg B (2000). The euglycemic hyperinsulinemic clamp examination: Variability and reproducibility. Scand. J. Clin. Lab Invest..

[14] Ferrannini E, Mari A (1998). How to measure insulin sensitivity. J. Hypertens..

[15] Bergman RN, Finegood DT, Ader M (1985). Assessment of insulin sensitivity in vivo. Endocr. Rev..

[16] Chen H, Sullivan G, Quon MJ (2005). Assessing the predictive accuracy of QUICKI as a surrogate index for insulin sensitivity using a calibration model. Diabetes.

[17] Legro RS, Finegood D, Dunaif A (1998). A fasting glucose to insulin ratio is a useful measure of insulin sensitivity in women with polycystic ovary syndrome. J. Clin. Endocrinol. Metab..

[18] McAuley KA, Williams SM, Mann JI (2001). Diagnosing insulin resistance in the general population. Diabetes Care.

[19] Park B, Jung DH, Lee HS, Lee YJ (2021). Triglyceride to HDL-cholesterol ratio and the incident risk of ischemic heart disease among Koreans without diabetes: A longitudinal study using national health insurance data. Front. Cardiovasc. Med..

[20] Krawczyk M, Rumińska M, Witkowska-Sędek E, Majcher A, Pyrżak B (2018). Usefulness of the triglycerides to high-density lipoprotein cholesterol ratio (TG/HDL-C) in prediction of metabolic syndrome in polish obese children and adolescents. Acta Biochim. Pol..

[21] Anoop S, Jebasingh FK, Rebekah G (2020). The triglyceride/glucose ratio is a reliable index of fasting insulin resistance: Observations from hyperinsulinaemic-euglycaemic clamp studies in young, normoglycaemic males from Southern India. Diabetes Metab. Syndr..

[22] Anoop SS, Dasgupta R, Rebekah G (2021). Lipid accumulation product (LAP) as a potential index to predict risk of insulin resistance in young, non-obese Asian Indian males from Southern India: Observations from hyperinsulinemic-euglycemic clamp studies. BMJ Open Diabetes Res. Care.

[23] Uwaifo GI, Fallon EM, Chin J, Elberg J, Parikh SJ, Yanovski JA (2002). Indices of insulin action, disposal, and secretion derived from fasting samples and clamps in normal glucose-tolerant black and white children. Diabetes Care.

[24] Florkowski CM (2008). Sensitivity, specificity, receiver-operating characteristic (ROC) curves and likelihood ratios: Communicating the performance of diagnostic tests. Clin. Biochem. Rev..

[25] Monaghan TF, Rahman SN, Agudelo CW (2021). Foundational statistical principles in medical research: Sensitivity, specificity, positive predictive value, and negative predictive value. Medicina (Mex).

[26] Hanley JA, McNeil BJ (1982). The meaning and use of the area under a receiver operating characteristic (ROC) curve. Radiology.

